# Supplementary figures and images for: A modified FASP protocol for high-throughput preparation of protein samples for mass spectrometry
Source: PLoS One. 2017 Jul 27;12(7):e0175967. doi: 10.1371/journal.pone.0175967 (PMC5531558; doi:10.1371/journal.pone.0175967)

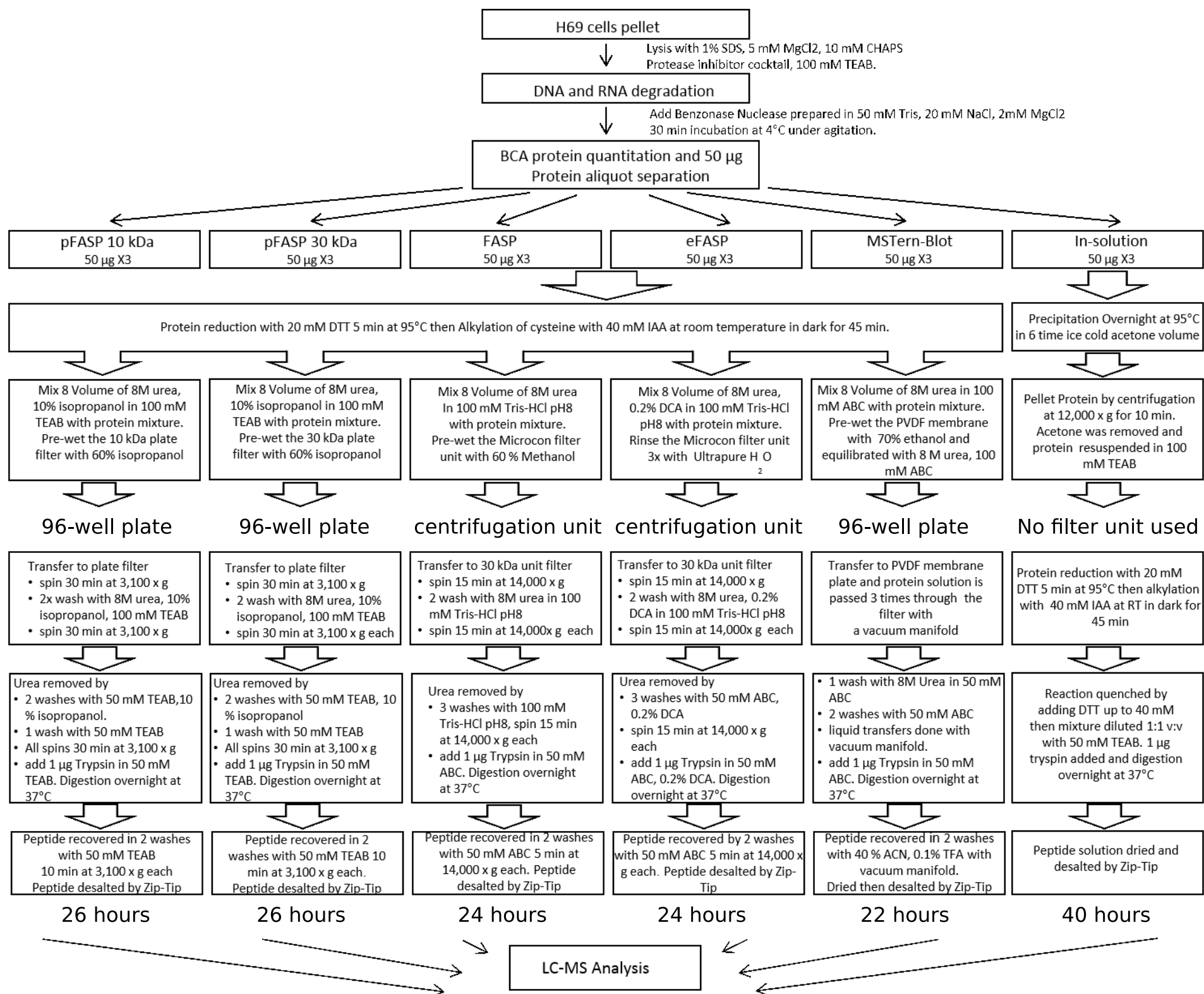

Supplement: S1 Fig — Preparation time per sample, including 18 hours of trypsin digest and overnight precipitation for the in-solution preparation, is indicated at the bottom of the flow chart. The preparation times show that pFASP is comparable to FASP-like methods that use centrifugation units. This allows for rapid, high-throughput processing of large numbers of samples in 96-well plates wih processing times similar to those of the FASP-like methods using individual centrifugation units. (PDF) [file pone.0175967.s001.pdf]
